# Cytocompatible and Antibacterial Properties of Chitosan-Siloxane Hybrid Spheres

**DOI:** 10.3390/polym11101676

**Published:** 2019-10-14

**Authors:** Yuki Shirosaki, Manato Nakatsukasa, Saki Yasutomi, Susana Cruz-Neves, Satoshi Hayakawa, Akiyoshi Osaka, Toshinari Maeda, Toshiki Miyazaki

**Affiliations:** 1 Faculty of Engineering, Kyushu Institute of Technology, 1-1 Sensui-cho, Tobata-ku, Kitakyushu, Fukuoka 804-8550, Japan; s-yasutomi@che.kyutech.ac.jp; 2 Graduate School of Natural Science and Technology, Okayama University, 3-1-1 Tsushima-naka, Kita-ku, Okayama 700-8530, Japan; en20746@s.okayama-u.ac.jp; 3 Graduate School of Life Science and Systems Engineering, Kyushu Institute of Technology, 2-4 Hibikino, Wakamatsu-ku, Kitakyushu, Fukuoka 808-0196, Japan; susanacruzneves@gmail.com (S.C.-N.); toshi.maeda@life.kyutech.ac.jp (T.M.); tmiya@life.kyutech.ac.jp (T.M.); 4 Graduate School of Interdisciplinary Science and Engineering in Health Systems, Okayama University, 3-1-1 Tsushima-naka, Kita-ku, Okayama 700-8530, Japan; satoshi@cc.okayama-u.ac.jp; 5 Faculty of Engineering, Okayama University, 3-1-1 Tsushima-naka, Kita-ku, Okayama 700-8530, Japan; akiosaka@okayama-u.ac.jp

**Keywords:** chitosan-siloxane hybrid, microporous spheres, cell behavior, cerium ion, antibacterial property

## Abstract

Microporous spheres in a hybrid system consisting of chitosan and γ-glycidoxypropyltrimethoxysilane (GPTMS) have advantages in a range of applications, e.g., as vehicles for cell transplantation and soft tissue defect filling materials, because of their excellent cytocompatibility with various cells. In this study, microporous chitosan-GPTMS spheres were prepared by dropping chitosan-GPTMS precursor sols, with or without a cerium chloride, into liquid nitrogen using a syringe pump. The droplets were then freeze dried to give the pores of size 10 to 50 μm. The cell culture tests showed that L929 fibroblast-like cells migrated into the micropores larger than 50 μm in diameter, whereas MG63 osteoblast-like cells proliferated well and covered the granule surfaces. The spheres with cerium chloride showed antibacterial properties against both gram-negative and gram-positive bacteria.

## 1. Introduction

Microporous spheres have advantages in a range of applications, e.g., as vehicles for transplantation of cultured cells and materials for filling defects in human tissues [1,2]. The pore size has important effects on cell behaviors such as adhesion, migration, and distribution, and also affects the exchange of nutrients and metabolite wastes. Shirosaki et al. reported that chitosan-γ-glycidoxypropyltrimethoxysilane (GPTMS) porous bulk hybrids had excellent cytocompatibility with various cells [3,4,5]. However, it is initially difficult for the cells to migrate into porous bulk hybrids [6]. Porous granules can form three-dimensional (3D) accumulations with openings into which the cells can migrate. In accumulations of porous granules, the approximate sizes of the circular gaps between the granules, as well as the pore size, influence the cell behavior. The porous granules of diameter 2 mm give circulate gaps of diameter of approximately 310 μm. This space size is suitable for osteoconduction [7].

Chitosan is widely used in antibacterial applications [8,9]. Positively charged chitosan binds to the negatively charged bacterial surface, leading to altered membrane permeability and the leakage of intracellular constituents, causing cell death. Generally, chitosan amino groups are protonated at low pH (pH < 6.0) and crosslinking decreases the number of free amino groups. However, in the human body, the pH is generally neutral. Chitosan swell easily in the wet condition without the crosslinking and have difficulty in maintaining their shapes. Some antibacterial drugs are therefore incorporated into a chitosan matrix and released by controlled degradation of the matrix. The degradation of chitosan-GPTMS hybrids can be controlled by crosslinking GPTMS [6,10]. This study focused on cerium as the antibacterial drug for the incorporation into chitosan-GPTMS hybrids. Cerium is a rare-earth element of the lanthanide group and has antibacterial properties [11]. In particular, cerium nitrate [12,13] and cerium oxide [14,15] have been used as antibacterial reagents. The uptake of cerium ions into cytoplasm can inhibit cellular respiration, oxygen uptake, glucose metabolism, and disrupt the cell membrane [16,17]. The incorporation of cerium ions into chitosan-GPTMS hybrids can compensate for the limitations in antibacterial properties by crosslinking amino groups.

In this study, microporous chitosan-GPTMS hybrid spheres were prepared by dropping hybrid precursor sols into liquid nitrogen using a syringe pump and then freeze drying the droplets. The effects of the micropore diameter on cell adhesion and migration were examined for MG63 osteoblast-like cells and L929 fibroblast-like cells. Additionally, cerium chloride was incorporated into the hybrid spheres and their antibacterial properties against *Escherichia coli* (gram-negative) and *Staphylococcus aureus* (gram-positive) were investigated.

## 2. Materials and Methods

### 2.1. Preparation of Chitosan-Siloxane Hybrid Spheres without/with Cerium Ions

**Figure 1:**
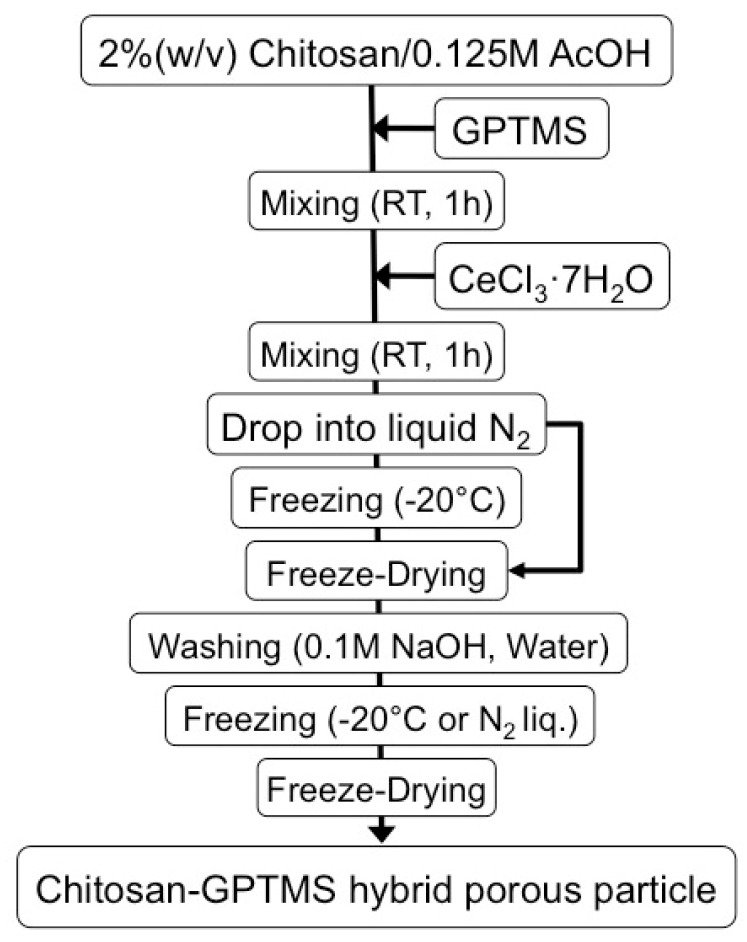
Preparation scheme of the chitosan-siloxane hybrid spheres.

Figure 1 shows the preparation scheme of the hybrid spheres. Chitosan powder (310,000–375,000 Da, DA > 75%; Sigma-Aldrich, St. Louis, MO, USA) was dissolved into 0.25 M aqueous acetic acid (AcOH) using a planetary centrifugal mixer (ARE-310, Thinky, Tokyo, Japan) to obtain 2 *w*/*v*% chitosan solution. The appropriate GPTMS (97%, Alfa Aesar, Heysham, UK) was stirred to be hydrolyzed in 0.25 M AcOH at room temperature for 1 h. The GPTMS/AcOH solution was added to the chitosan solution and the mixture was stirred at room temperature for 1 h. Cerium chloride heptahydrate (CeCl_3_·7H_2_O, Nacalai Tesque, Kyoto, Japan)/AcOH was added to the chitosan-GPTMS precursor sol. The final mixture was stirred again at room temperature for 1 h. The compositions with the sample codes are given in Table 1. To fabricate the spheres, the final mixture was dropped into liquid nitrogen using a syringe (27G) at a rate of 0.04 mL/min (Video S1). The iced droplets were kept in liquid nitrogen or at −20 °C for 1 d, transferred to a freeze dryer (FDU-1200, EYELA, Tokyo, Japan), and then lyophilized to complete dryness. After drying, the porous spheres were soaked in 0.1 M NaOH aqueous solution to neutralize the remaining acetic acid, washed with distilled water, and lyophilized again in the freeze dryer. The spheres were sterilized with ethylene gas and kept for 1 week at room temperature to clear any remaining ethylene oxide gas.

### 2.2. Characterization of Chitosan-Siloxane Hybrid Spheres

The 3D X-ray micro-CT scans were performed using a TDM 1000 H-II (2k) system (Yamato Scientific Co., Tokyo, Japan) with a 35 kV (0.1 mA) microfocus X-ray source at the Microscopic Scan Co. (Tokyo). The spheres were placed inversely on a polystyrene foam cube inserted between the sails and the rotating stage, and were adhered to the cube with double-sided tape. The samples were scanned in an atmospheric environment. The X-ray shadow images were acquired in 1000 views, at 15 frames per view, with a pixel resolution of 0.5 to 8 μm (approximately 20 min scan). The X-ray shadow images were reconstructed into 3D cross-sections using the VGStudio MAX 2.1 program (Volume Graphics GmbH, Heidelberg, Germany), and all subsequent analyses were based on the volume data set. A cross-sectional two-dimensional image was obtained at equally spaced planes (center) of the sample. The surface morphology was examined using scanning electron microscopy (SEM; JMS-6010 PLUS/LA, JEOL, Tokyo, Japan) with energy dispersive X-ray spectroscopy (EDX). Before the observations, the spheres were coated with Pt/Pd of thickness of approximately 20 nm (MSP-1S Magnetron Sputter, Vacuum Device Inc., Mito, Japan). The mean pore diameter was obtained from the SEM images using ImageJ v1.48 software (National Institutes of Health, Bethesda, MD, USA). At least 20 pores were assessed from three different areas of the same sample.

The degree of crosslinking was evaluated using a ninhydrin assay (n = 3), and defined as the percentage of free amino groups in the spheres [18]. The spheres were suspended in the ninhydrin buffer solution (ninhydrin reagent L-8500 Set, Wako Pure Chemical Industries, Ltd., Osaka, Japan). The ninhydrin reagent was added and then the mixture was kept at 80 °C for 20 min. The optical absorbance of the supernatant solution was recorded at 570 nm using a spectrophotometer (DS11+, DeNovix Inc., Wilmington, DE, USA). The relative percentages of free amino groups in the chitosan-GPTMS-cerium spheres toward the chitosan only spheres were calculated.

### 2.3. In Vitro Cytocompatibility Tests

The ChG05 spheres prepared at different temperatures and treated with NaOH were used in cell culture tests because the ChG05 hybrid improved the differentiation of human bone marrow cells [5]. The osteoblast-like cells (MG63) and fibroblast-like cells (L929) were cultured and then cell suspensions containing 5.0 × 10^4^ cells/300 μL medium were prepared. Dulbecco’s modified Eagle’s medium (MEM) supplemented with 10 vol% fetal bovine serum (FBS) was used for MG63 and MEM supplemented with 10 vol% FBS and 0.03% L-glutamine was used for L929. Seven spheres were placed in 96-well plates and a cell suspension (300 μL) was added to each plate. After incubation for 7 and 14 d at 36.5 °C in a humidified atmosphere consisting of 5% CO_2_ in air, the spheres with the cells were transferred to a new plate and fixed in a 1.5% glutaraldehyde/0.14 M sodium cacodylate buffer (pH 7.3), and then dehydrated in graded ethanol and *t*-butanol. After dehydration, the spheres were freeze dried at 13.3 Pa and −5 °C using a freeze-dryer (JFD-310, JEOL, Tokyo, Japan). The spheres were coated with a thin gold film and observed using SEM. Some spheres were stained with haematoxylin-eosin and observed using an optical microscope.

### 2.4. In Vitro Antibacterial Property Tests

To inhibit the burst release of cerium ions and activate the antibacterial properties of the amino groups, ChG10 spheres prepared at −196 °C without NaOH treatment were used for the antibacterial property tests. *E. coli* NIHJ and *S. aureus* 209P were suspended in Luria-Bertani Broth medium (Liofilchem, TE, Italy). The optical density of the bacterial suspension was adjusted to 1.0 (1 × E^2^ CFU/mL). Five spheres were placed in 96-well plates and bacterial suspension (200 μL) was added to each plate. The bacterial viability after contact with the spheres was determined using the 3-[4,5-dimethylthiazol-2-yl]-2,5-diphenyltetrasodium bromide (MTT) assay (*n* = 5). MTT is reduced to a purple formazan reaction product by living cells and bacteria [19]. After incubation at 37 °C for 24 h, MTT (20 μL) was added. The spheres were incubated at 37 °C for 4 h, and then transferred to a new plate. The formazan salts were dissolved in dimetylsulfoxide (200 μL) and the absorbance at 600 nm were measured to evaluate the bacterial viability. The pH changes by the spheres were also monitored using phosphate-buffered saline (PBS).

## 3. Results and Discussion

### 3.1. Sphere Micromorphologies

**Figure 2:**
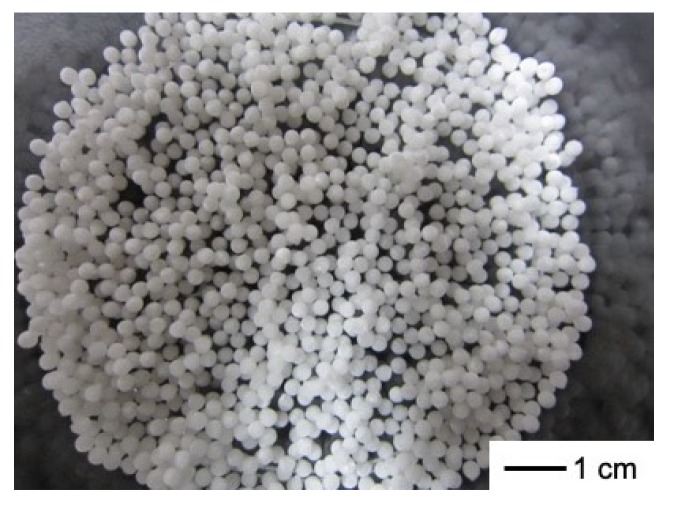
Appearance of chitosan-siloxane hybrid spheres (ChG05, −20 °C).

**Figure 3:**
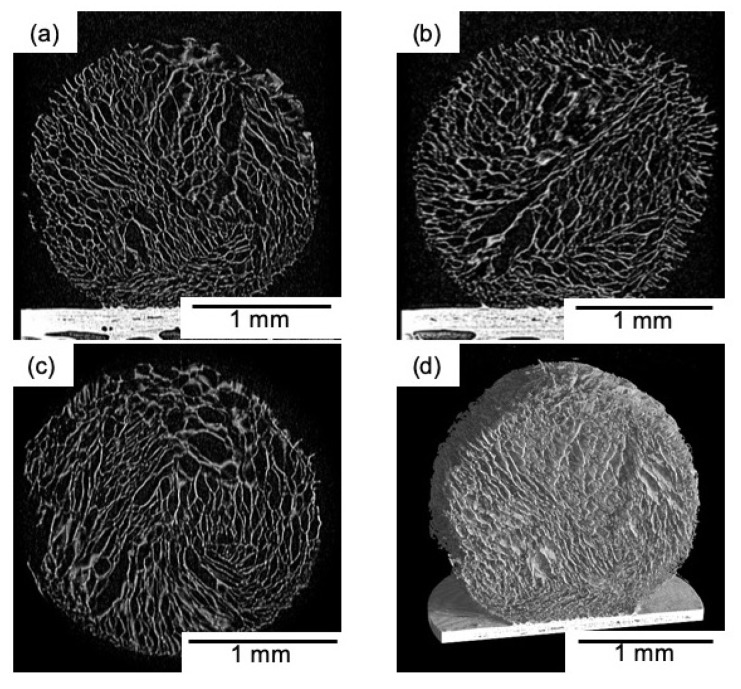
Transverse plane, (**a**) frontal slice (x,z), (**b**) sagittal slice (y,z), (**c**) axial slice (x,y) and 3D reconstruction (**d**) images of chitosan-siloxane hybrid spheres (ChG05, −20 °C) obtained using micro-CT scan.

**Figure 4:**
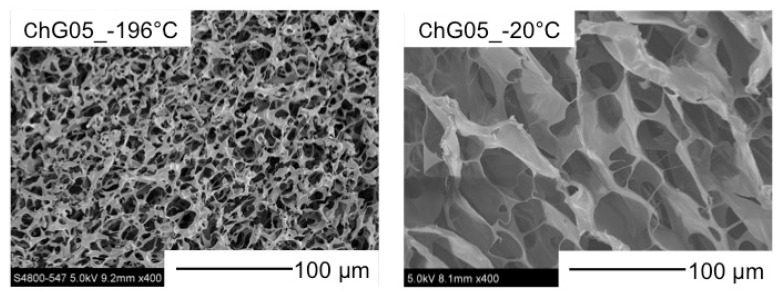
SEM images of surfaces of ChG05 chitosan-siloxane hybrid spheres prepared at −20 and −196 °C.

Figure 2 shows that the spheres are of homogeneous size. The sphere’s diameter was 2 mm, regardless of the freezing temperature and composition. The porous structures were interconnected (Figure 3) and the pore sizes depended on the freezing temperature. Table 2 and Figure 4 show that the pore size of the spheres frozen at −196 °C (10 μm) is one-fifth of those frozen at −20 °C (50 μm). In freeze drying, the pore sizes are determined by the ice crystal size [20]. At a lower freezing temperature, the initial number of ice nuclei is larger than that at a higher temperature, leading to a decrease in the final size of the ice crystals.

### 3.2. Cell Proliferation and Migration

**Figure 5:**
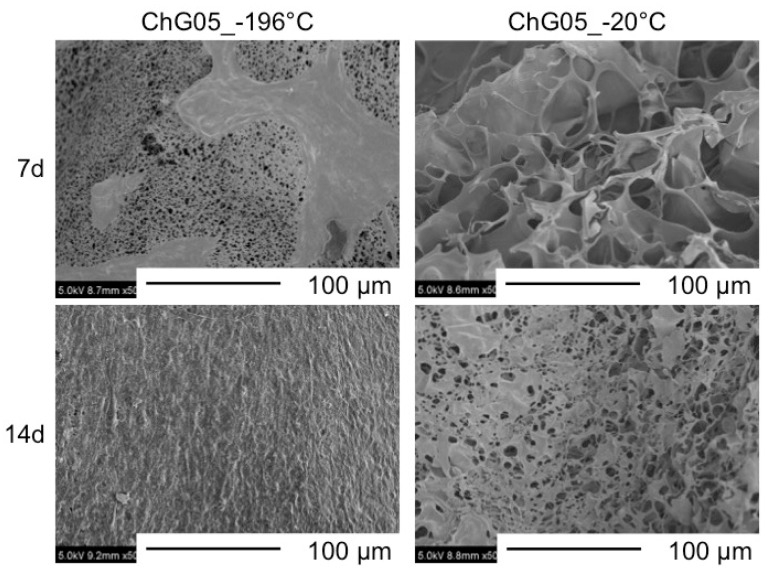
SEM images of MG63 cells cultured on surfaces of ChG05 chitosan-siloxane hybrid spheres prepared at −20 and −196 °C.

**Figure 6:**
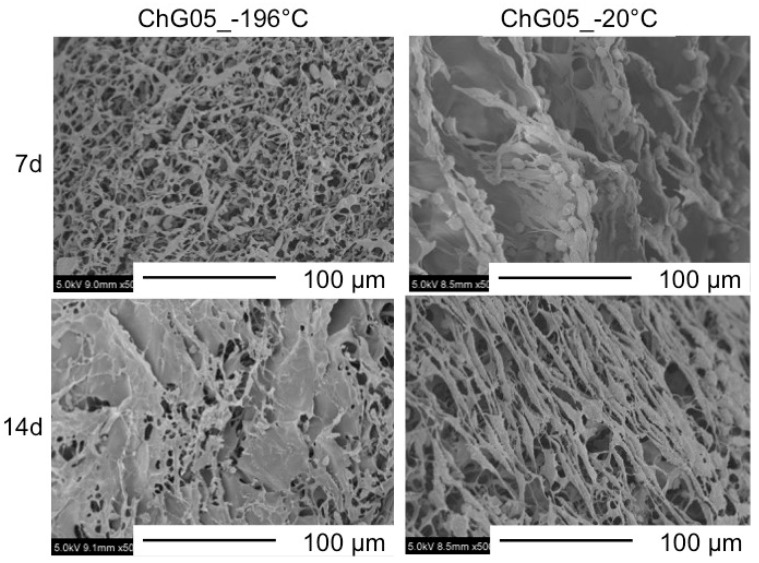
SEM images of L929 cells cultured on surfaces of ChG05 chitosan-siloxane hybrid spheres prepared at −20 and −196 °C.

**Figure 7:**
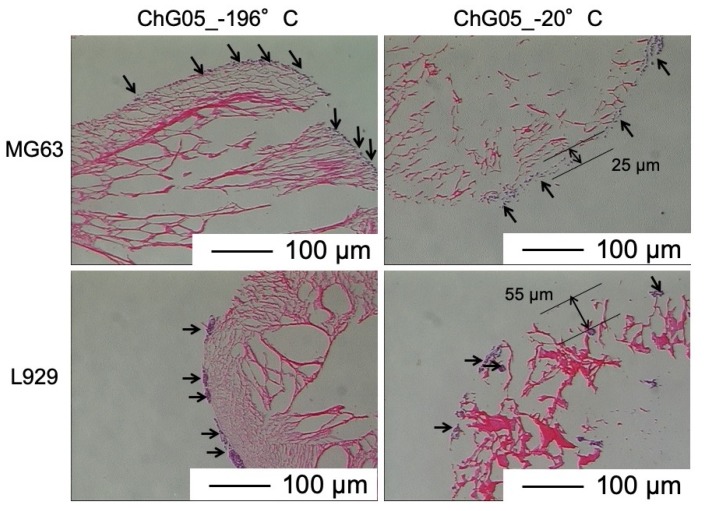
Haematoxylin-eosin staining of L929 and MG63 cells cultured for 14 d on surfaces of ChG05 chitosan-siloxane hybrid spheres prepared at −20 and −196 °C. Dark purple with arrows showed cells.

The authors have previously reported on the biodegradability of the porous hybrid with the same compositions in a lysozyme solution [21]. The weight loss of ChG05 was approximately 2% after 1 month of soaking. This means that the chitosan-siloxane matrix structure is very stable in the medium. Figure 5 and Figure 6 show SEM images of MG63 and L929 cells cultured on ChG05 spheres prepared at −196 °C and −20 °C. The MG63 and L929 cells proliferated on both typed of spheres. The MG63 cells hardly migrated into the micropores of a diameter of approximately 10 µm, but migrated into the micropores of a diameter of approximately 50 µm. The haematoxylin-eosin stained images in Figure 7 show that the migration distance is approximately 25 µm. In the case of L929, the number of migrating cells and the migration distance were higher than in the case of MG63, even for approximately 10 μm micropores. The L929 cell migration distance was approximately 55 µm. The research on the optimum pore size ranges for different types of cells or tissues has shown that the fibroblasts favor smaller pores [22] compared with osteoblast cells [7,23]. The L929 cells migrated more than the MG63 cells, even into the micropores of diameter of less than 50 µm. After 14 d, both types of cells entirely covered the sphere surfaces, and the cells combined well with each other. The spheres with micropores of diameter of approximately 10 µm prepared at −196 °C had large specific surface areas. This is important for initial cell adhesion and improves cell proliferation. After covering the surfaces, the cells and spheres can accumulate to form 3D scaffolds because the size of the spaces between the spheres is favorable for the cells.

### 3.3. Antibacterial Properties of Spheres with Cerium Chloride

**Figure 8:**
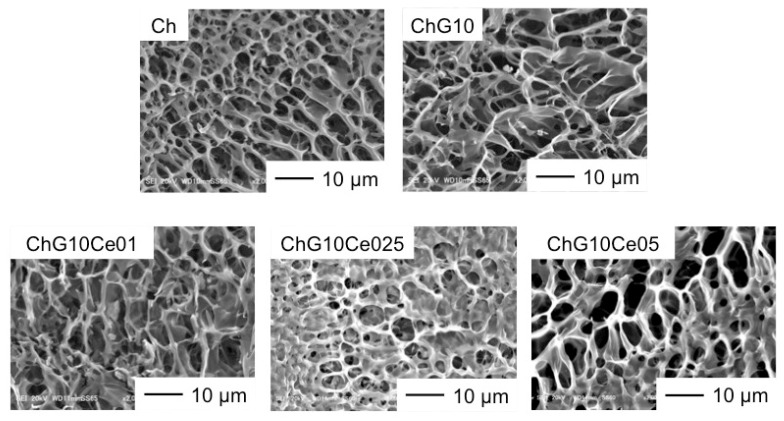
SEM images of surfaces of Ch chitosan and ChG10 chitosan-siloxane hybrid spheres with various amounts of incorporated cerium chloride.

**Figure 9:**
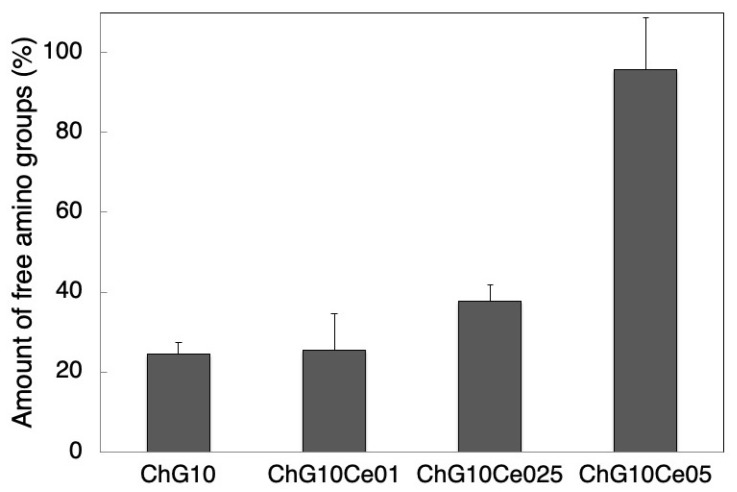
Percentages of free amino groups by ninhydrin reaction in ChG10 chitosan-siloxane hybrid spheres with various amounts of incorporated cerium chloride.

**Figure 10:**
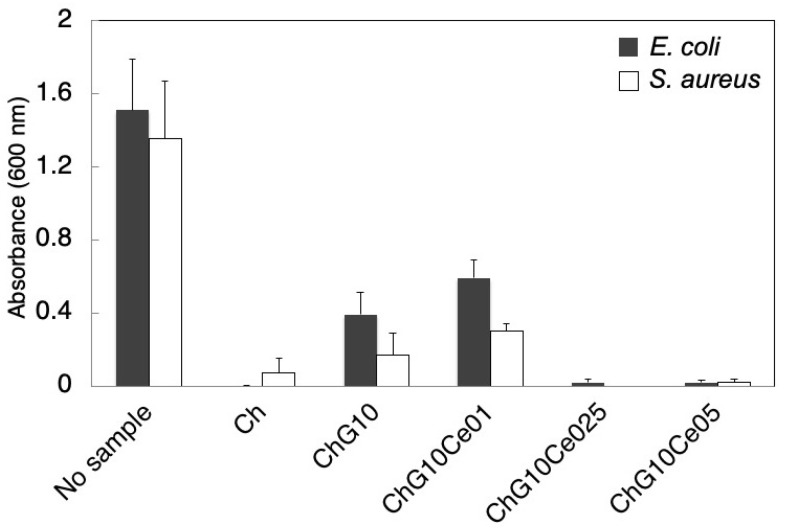
Viabilities of *E. coli* and *S. aureus* cultured for 24 h with Ch chitosan and ChG10 chitosan-siloxane hybrid spheres with various amounts of incorporated cerium chloride.

To activate the antibacterial properties of the free amino groups of chitosan, the spheres without NaOH treatment were used for the antibacterial property tests. Figure 8 shows SEM images of ChG10 prepared using different amounts of cerium chloride. The pore sizes are summarized in Table 2. The ChG10 pores were smaller than the ChG05 pores because of crosslinking between amino groups and epoxide groups, and polycondensation of siloxane units [4,5]. The incorporation of cerium chloride did not have an effect on the pore size. The ionic radius of trivalent cerium (1.01 Å) and bivalent calcium (1.00 Å) are very similar [11] and show similar chemical behavior. Two amino groups of chitosan form complexes with calcium ions [24]. In this study, the crosslinking of amino groups of chitosan was inhibited which means that cerium ions interacted with amino groups. Further, the epoxide groups of GPTMS cannot react with chitosan. This is supported by the percentages of free amino groups shown in Figure 9. In ChG10, GPTMS was crosslinked with 75% of the chitosan amino groups. The additional of cerium chloride to chitosan-GPTMS increased the amount of free amino groups. The EDX results (Table 3) show that the proportion of cerium on the sphere surfaces increased with the increasing amount of incorporated cerium chloride. Figure 10 shows the viabilities of *E. coli* and *S. aureus* cultured with the spheres for 24 h. The PBS pH values after soaking the spheres are listed in Table 3. The bacterial viabilities with the spheres showed lower than that without spheres. Non-crosslinked chitosan (Ch) spheres have antibacterial properties as previously reported [8,9,25,26,27] at lower than pH 6.5. The amino groups are positively charged below pH 6 and interact with negatively charged microbial cell membranes, leading to protein leakage [8,9,28], because of the hydrolysis of peptidoglycans in the walls and changes in the properties of cell membrane permeability. In our experiments, the pH of the medium was not so low (pH 6.8 after 24 h), however, the free amino groups of Ch were sufficiently protonated to cause cell death. Crosslinking with GPTMS lost the antibacterial properties of chitosan because the free amino groups were insufficient. ChG10Ce0.1 and ChG10 had the same amount of free amino group on their surfaces and were insufficient to inhibit bacterial growth. This means that a small amount of cerium cannot inhibit crosslinking between amino groups of chitosan and epoxide groups of GPTMS. On the other hand, ChG10Ce025 and ChG10Ce05 with an increased amount of cerium, killed bacteria. In particular, the amino groups of ChG10Ce05 were completely free and inhibited the crosslinking of amino groups with epoxide groups by cerium ions to manifest anti-bacterial properties. The free amino groups of ChG10Ce025 were only 38%, but cerium of the sphere surfaces also had the effect on the bacterial viability. Ch completely lost their spherical shapes and ChG10Ce05 swelled during soaking in the cell suspension. The results suggest that a cerium molar ratio of 0.25–0.5 is the optimum amount for antibacterial spheres. Our preliminary tests show that the minimum inhibitor concentration (MIC) of cerium chloride for both bacteria was 3 mM (Figure S1). The released cerium chloride from ChG10Ce025 can be estimated to 1.7 mM in the medium and it is lower than MIC. Chitosan is hydrophilic to kill gram-positive bacteria (*S. aureus*) and gram-negative bacteria (*E. coli*) [9,25,28]. Gram-negative bacteria, such as *E. coli*, have an outer membrane that surrounds the cell wall, preventing drugs from interacting with the peptidoglycan that makes up the cell wall [29]. However, gram-positive bacteria, such as *S. aureus*, do not have this outer membrane and their cell walls remain vulnerable. In this study, the chitosan-siloxane spheres showed antibacterial activity against both *S. aureus* and *E. coli*. Bivalent ions, such as Mg^2+^ and Ca^2+^ were transported into the bacterial through the lipopolysaccharides (LPS) in the outer membrane [30]. The LPS confused Ce^3+^ with Ca^2+^ and was transported into the outer membrane, and resulted in up-taking it into the cytoplasm to inhibit the cellular respiration, glucose metabolism and membrane disruption [16].

## 4. Conclusions

Microporous chitosan-GPTMS hybrid spheres were prepared using liquid nitrogen and a freeze-drying system. The osteoblast-like cells and fibroblast-like cells covered the sphere surfaces and the fibroblast-like cells migrated into the micropores of size 50 μm. The spheres with the cells could accumulated to form 3D scaffolds at the tissue defects, resulting in regeneration. The spheres with cerium chloride showed antibacterial effects against both gram-negative and gram-positive bacteria. The free amino groups and cerium ions of spheres expressed the antibacterial properties at the acidic condition.

## Figures and Tables

**Table 1:** Starting compositions of spheres (molar ratio).

Sample	Chitosan	GPTMS	CeCl_3_
Ch	1.0	0	0
ChG05	1.0	0	0
ChG10	1.0	1.0	0
ChG10Ce01	1.0	1.0	0.1
ChG10Ce025	1.0	1.0	0.25
ChG10Ce05	1.0	1.0	0.5

**Table 2:** Pore sizes of the chitosan-siloxane spheres.

Sample	Freezing Temperature (°C)	Pore Size (μm)
Ch	−196	5.6 ± 13.6
ChG05	−20	48.6 ± 16.5
ChG05	−196	10.3 ± 3.7
ChG10	−196	5.9 ± 5.2
ChG10Ce01	−196	6.2 ± 5.1
ChG10Ce025	−196	5.6 ± 2.2
ChG10Ce05	−196	6.6 ± 3.7

**Table 3:** Cerium/carbon atomic ratios on spheres surfaces obtained using EDX and pH values of PBS after soaking spheres for 24 h.

Sample	Ce/C	pH
Ch	0	6.8
ChG10	0	6.6
ChG10Ce01	0.007	6.1
ChG10Ce025	0.019	5.8
ChG10Ce05	0.032	5.9

## Supplementary Materials

The following are available online at https://www.mdpi.com/2073-4360/11/10/1676/s1. Video S1: Movie of dropping the precursor sols into liquid N_2_ (2.0× Speed). Figure S1: Viability of *E. coli* and *S. aureus* in the medium with different concentration of cerium chloride.
