# Supplementary figures and images for: Cytocompatible and Antibacterial Properties of Chitosan-Siloxane Hybrid Spheres
Source: Polymers (Basel). 2019 Oct 14;11(10):1676. doi: 10.3390/polym11101676 (PMC6835879; doi:10.3390/polym11101676)

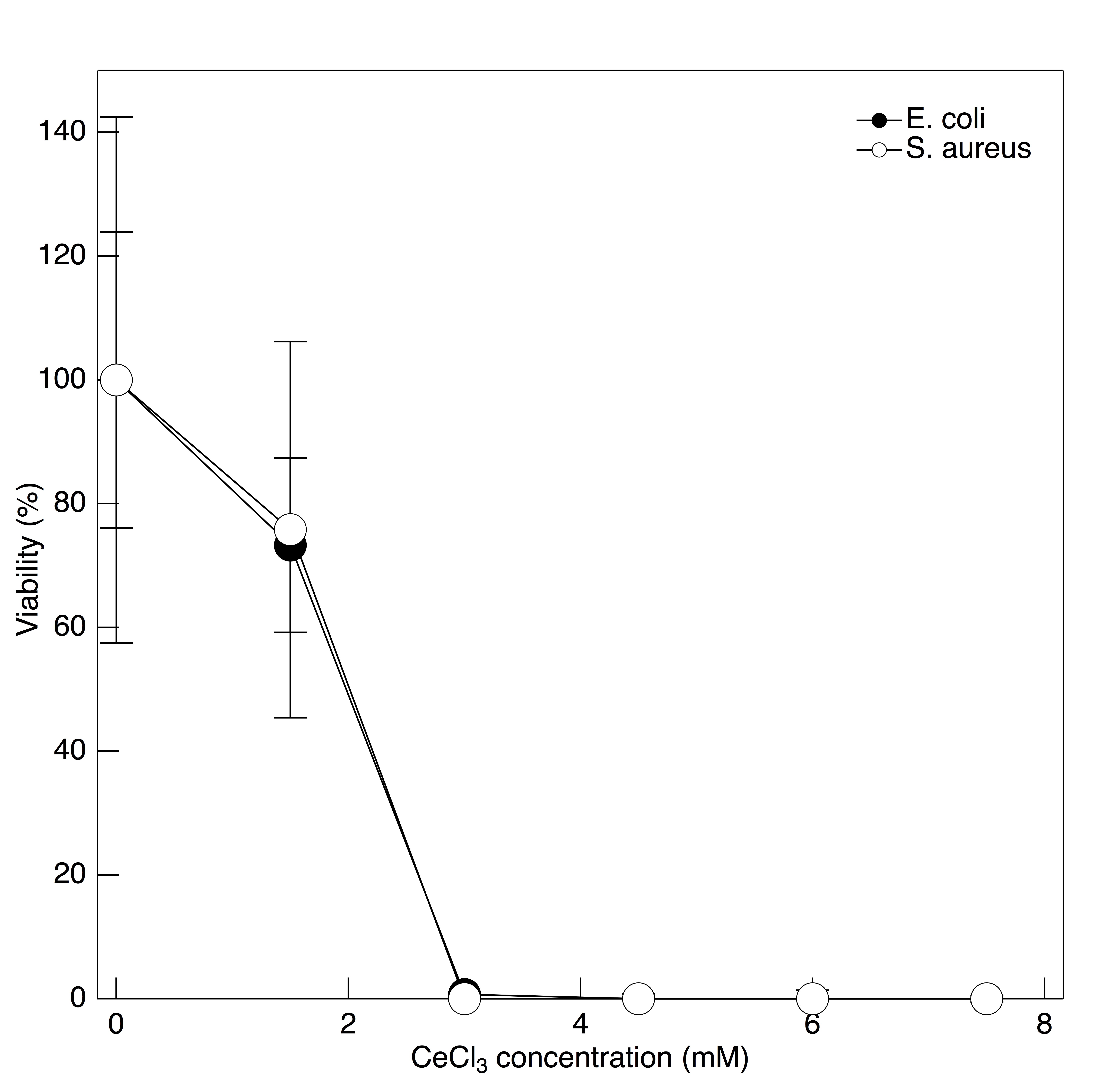

Supplement: Supplementary file 1 [file polymers-11-01676-s001.zip › Supplement/Dupplement_Figure1.jpg]
